# Incorporation of non-canonical amino acids into the developing murine proteome

**DOI:** 10.1038/srep32377

**Published:** 2016-08-30

**Authors:** Sarah Calve, Andrew J. Witten, Alexander R. Ocken, Tamara L. Kinzer-Ursem

**Affiliations:** 1 Weldon School of Biomedical Engineering, Purdue University, West Lafayette, IN 47907, USA.

## Abstract

Analysis of the developing proteome has been complicated by a lack of tools that can be easily employed to label and identify newly synthesized proteins within complex biological mixtures. Here, we demonstrate that the methionine analogs azidohomoalanine and homopropargylglycine can be globally incorporated into the proteome of mice through facile intraperitoneal injections. These analogs contain bio-orthogonal chemical handles to which fluorescent tags can be conjugated to identify newly synthesized proteins. We show these non-canonical amino acids are incorporated into various tissues in juvenile mice and in a concentration dependent manner. Furthermore, administration of these methionine analogs to pregnant dams during a critical stage of murine development, E10.5–12.5 when many tissues are assembling, does not overtly disrupt development as assessed by proteomic analysis and normal parturition and growth of pups. This successful demonstration that non-canonical amino acids can be directly administered *in vivo* will enable future studies that seek to characterize the murine proteome during growth, disease and repair.

Significant advances in proteomics technology have been made over the past decades facilitating the identification and quantification of proteins within complex biological samples. In particular, the bottom-up approach in which proteins are digested into peptides and analyzed using a combination of chromatographic separation, mass spectrometry and automated database matching of peptides has greatly increased the volume of data that can be used to describe complex systems^1^. However, there are only few studies that have employed these techniques to assess protein turnover during embryogenesis^2^,^3^,^4^,^5^.

As many developmental biologists are interested in what happens during a defined stage, it is critical to be able to specifically label proteins that are synthesized within a narrow time window. One way in which MS-based analysis can reveal synthesis and turnover rates is through stable isotope tracer incorporation (SILAC)^6^,^7^. Utilization of this method for proteomic analysis of adult and embryonic murine tissues was recently demonstrated^8^; however, it takes weeks and specialized chow to generate these mice. Another significant limitation of SILAC labeling is that proteins in low abundance can be missed^9^.

To overcome the drawbacks of current proteomic methods, and to create a means for selective enrichment of newly synthesized proteins, bioorthogonal non-canonical amino acid labeling (BONCAT) labeling was developed^10^. The Met analogs azidohomoalanine (AHA) and homopropargylglycine (HPG) carry azide and alkyne functional groups, respectively (Fig. 1A), and are incorporated into sites normally occupied by Met during protein synthesis (Fig. 1B)^11^. Importantly, these functional groups are bioorthogonal; they do not cross react with natural biological chemistries. Azides can react specifically with alkynes using copper-catalyzed azide-alkyne cycloaddition (CuAAC)^12^, allowing for the selective targeting of proteins containing AHA and HPG within complex mixtures of biomolecules (Fig. 1C). In this way AHA- and HPG-containing proteins can be reacted with fluorophores for imaging or affinity tags for enrichment^10^,^13^,^14^,^15^. The different functional groups of AHA and HPG make these reagents useful for pulse-chase experiments within the same system^16^.

AHA and HPG have been successfully utilized to label newly synthesized proteins *in vitro* and have proven to be nontoxic in zebrafish and *Xenopus in vivo*^13^,^17^,^18^. It had been hypothesized that global *in vivo* labeling of newly synthesized murine proteins using AHA and HPG would be infeasible due to the 390–500-fold higher affinity of methionine for the Met tRNA^19^,^20^,^21^. However, recent studies showed local incorporation from intraocular injections in the rat and global incorporation by feeding mice a specialized chow in which methionine was replaced with AHA^22^,^23^. Here, we extend these studies by demonstrating that AHA and HPG can be globally incorporated into the proteome of mice through facile intraperitoneal injections. These non-canonical amino acids (ncAAs) are incorporated into all tissues investigated in juvenile mice and are robustly integrated into the embryo, without disrupting development, when injected into pregnant dams. Our results indicate that ncAA labeling is a promising new tool that can be used to characterize the dynamics of protein synthesis and turnover at particular time points during embryonic development.

## Results

### *In vivo* incorporation of AHA and HPG into murine tissues

**Figure 1:**
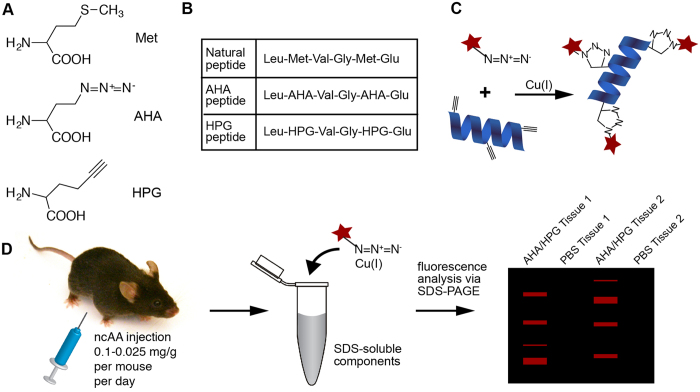
Metabolic labeling with non-canonical amino acids. (**A**) Amino acids in use in this study: Methionine (Met), Azidohomoalanine (AHA), Homopropargylglycine (HPG). (**B**) Peptides and proteins that naturally contain Met will instead be synthesized with AHA or HPG in the place of Met. (**C**) Newly synthesized proteins that have incorporated HPG (blue ribbon) are labeled with a fluorophore-conjugated azide (red star). Copper-catalyzed azide/alkyne cycloaddition results in a stable triazole adduct. (**D**) Mice were injected with varying amounts of ncAA for two days. Tissues were harvested, solubilized, reacted with azide- or alkyne-conjugated fluorophore then analyzed with SDS-PAGE.

**Figure 2:**
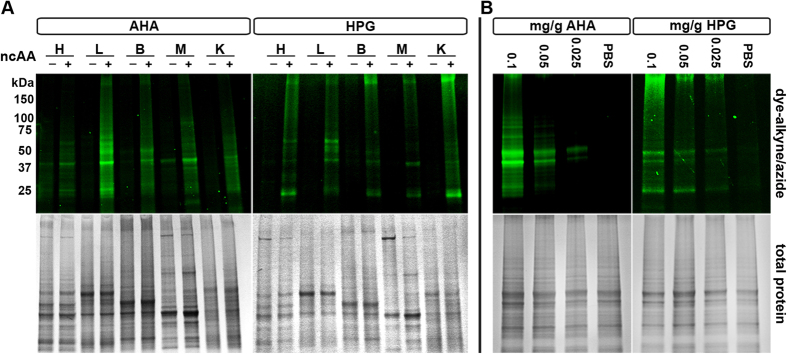
Systemic and dose-dependent incorporation of non-canonical amino acids (ncAA) in murine tissues. (**A**) Juvenile mice were injected IP with 0.1 mg/g per day with AHA, HPG or PBS for two days and heart (H), lung (L), brain (B), skeletal muscle (M) and kidney (K) lysates were compared. (**B**) Brain lysates from juvenile mice injected with 0.025–0.1 mg/g AHA, HPG or PBS. Tissue lysates were labeled with fluorophore-conjugated azide or alkyne, resolved using SDS-PAGE, fluorescently imaged then stained for total protein with Coomassie Blue to confirm equal loading. Representative results from N ≥ 3 independent experiments.

To establish the feasibility of BONCAT labeling *in vivo*, juvenile mice (25–35 days old; 14–20 g) were injected intraperitoneally (IP) with 0.1 mg/g per day AHA, HPG or vehicle alone (PBS; Fig. 1D). Tissue lysates were reacted with either a AF555-conjugated alkyne or azide using CuAAC and resolved using SDS-PAGE (Fig. 1D). All murine tissues investigated incorporated AHA and HPG (Fig. 2). In addition, the rate of AHA and HPG incorporation was dose dependent. Mice were injected with 0.1, 0.05 and 0.025 mg/g per day and there was a clear dosage-dependent increase in AHA and HPG incorporation (Fig. 2).

### Metabolic labeling of embryos *in situ*

**Figure 3:**
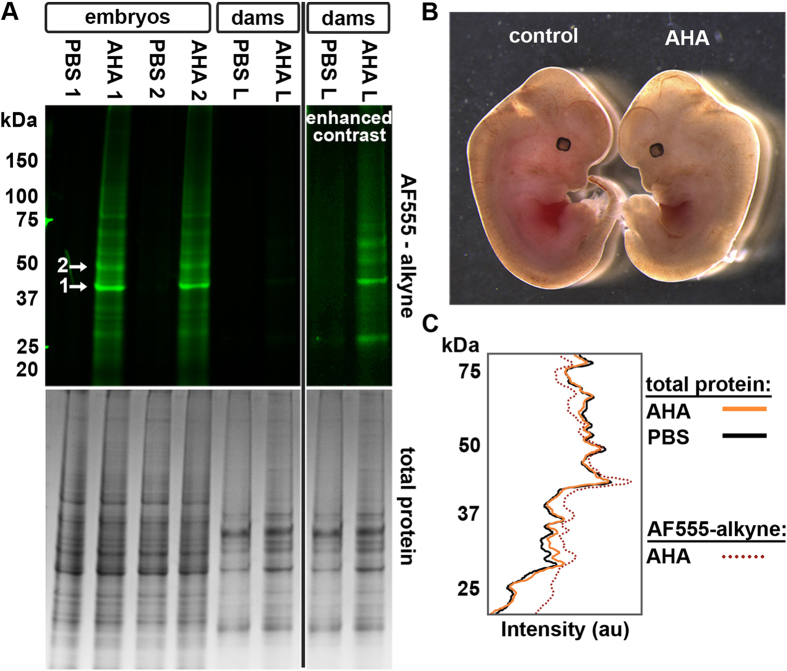
AHA is robustly incorporated into the developing murine proteome. (**A**) Dams injected with 0.1 mg/g per day for 2 days incorporated AHA into the developing embryos and their own tissues; however, the latter was much lower as indicated by the need for contrast enhancement (L = lung). Tissue lysates were labeled with fluorophore-conjugated alkyne, resolved using SDS-PAGE, fluorescently imaged then stained for total protein with Coomassie Blue to confirm equal loading. Representative results from N ≥ 3 independent experiments. Arrows indicate the bands excised for proteomics analysis described in Fig. 4. (B) Age matched embryos from control and AHA-injected dams show no difference in size and protein banding pattern (**A**). (**C**) Intensity tracings of the total protein bands in lanes 2 (AHA) and 3 (PBS) show that overall protein expression was not changed by AHA administration. Fluorescence intensity of proteins in lane 2 (dotted line) reveals that peaks occur in the same locations as in the total protein (solid lines). Representative results from N ≥ 3 pregnancies.

To test if methionine analogs could be used to label newly synthesized proteins in developing embryos, time-mated females were injected IP with 0.1 mg/g per day AHA or vehicle alone (PBS) for two days, starting at E10.5. Lysates of E12.5 embryos showed strong AHA incorporation compared with lungs from the dam (Fig. 3A). Embryos from AHA-injected dams were viable as indicated by a persistent heartbeat after removal from the uterus (Fig. 3B).

The intensity profiles of the total protein content of AHA and PBS samples were compared by line trace intensity analysis to qualitatively evaluate if protein expression was altered (Fig. 3C). There did not appear to be a substantial change in total protein expression as a result of AHA administration. To assess if AHA was homogenously incorporated into the proteome, a line trace (Fig. 3C) of the fluorophore-conjugated lysates was compared with the total protein (lane 2, Fig. 3A). While the baseline intensity of the line trace for the fluorescent and total protein was not consistent along the lane, the peak intensities occurred at the same molecular weights.

Additional dams injected with AHA between E10.5–12.5 were allowed to carry their litters to term. Parturition occurred when expected and P10 and P22 pups displayed the appropriate weight and physical markers (Fig. S1). As the pups matured, no behavioral or physical differences between AHA and control litters were noted (casual observation). Indeed, mice from AHA and control litters continued to develop as expected beyond 6th months (as of submission date). Overall, these data indicate that AHA administration does not perturb development during this time window in which many critical tissues are actively assembling (Fig. 3).

### AHA administration does not substantially change the proteome of developing mice

**Figure 4:**
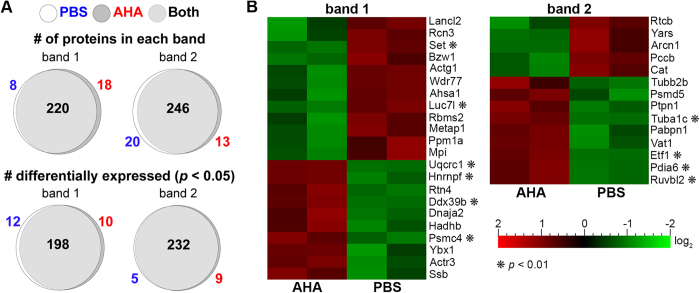
AHA administration minimally impacts the embryonic proteome. Two bands at ~42kDa and 48kDa with high levels of AHA labeling (arrows Fig. 3A) were digested in-gel and analyzed using LC-MS/MS. (**A**) The majority of proteins present in AHA and PBS samples were identified in both populations (top, Table S1). Of the proteins expressed in both AHA and PBS, only 5–10% showed significantly different levels of expression as analyzed using MaxQuant (*p* < 0.05, bottom). (**B**) Heat map indicating the identities and log2-fold difference in expression of all proteins differentially expressed in bands 1 and 2.

Biological replicates from PBS and AHA pregnancies were compared to determine if the proteome of the developing embryo was affected by 2 days of AHA treatment. Two bands corresponding to robust fluorescent labeling were chosen from AHA samples and the PBS controls (Fig. 3A), and analyzed using LC-MS/MS. Only proteins present in both technical replicates were considered for subsequent analysis. Of the 246 unique proteins found in band 1, 89% (220) were present in both the AHA and PBS samples, and only 10% of those 220 proteins showed significantly different levels of expression (*p* > 0.05; Fig. 4). Similarly, for band 2, 88% (246) of proteins were present in both the AHA and PBS samples, and only 5.7% showed significantly different levels of expression (*p* > 0.05; Fig. 4). When considering a more stringent threshold, *p > 0.01, the number of proteins in bands 1 and 2 that were significantly drops to 2.7% and 1.7%, respectively (Fig. 4B, Table S1).

## Discussion

Due to the low propensity of AHA and HPG for the Met tRNA compared with endogenous Met, it has been thought that it would not be feasible to supply enough for *in vivo* incorporation^19^,^20^,^21^. However, we demonstrate that AHA and HPG administered IP will globally incorporate into the proteome of juvenile mice and developing embryos.

To estimate how much more AHA than Met was in our test animals, we first calculated the total amount of Met in the body fluid of a mouse. The concentration of free Met in the plasma of C57BL6 mice ranges from 20–80 μM^24^,^25^. If it is assumed that the concentration of free Met is the same in all body fluids, and taking in to account that 58% of the weight of a mouse is water^26^, a 20 g mouse will be comprised of 11.6 mL of fluid that contains 50 μM Met. Using the MW of free Met (149.21 g/mol), there is approximately 4.4 μg free Met per gram of total mouse weight. Therefore, when injecting 0.1 mg/g, there will be 20-fold more AHA/HPG than Met. How AHA/HPG is metabolized, and the identity of the resultant degradation products, remains unknown and will warrant further investigation as these ncAAs become more widely implemented *in vivo*. Excess systemic ncAAs and their metabolites have the potential to induce pathological states similar to what has been shown with naturally occurring amino acids such as hyperhomocysteinemia (excess Met)^27^. Nevertheless, data from numerous *in vitro* and *in vivo* reports^9^,^10^,^13^,^17^,^18^, in addition to our *in vivo* demonstration that AHA does not disrupt mammalian development (Fig. 3), indicate that short term administration of ncAAs has minimal adverse effects.

Protein banding patterns between AHA/HPG and control tissue lysates were consistent, suggesting normal physiological function was maintained. It appears that HPG is incorporated to a lower extent than AHA, which correlates with previous findings *in vitro* that HPG charges onto the Met tRNA at a slower rate than AHA^21^. Overall, our data demonstrate the feasibility of systemic administration and labeling of other ncAAs that can be utilized by endogenous tRNA synthetases, such as homoallylglycine^28^.

When the proteomes of control and AHA-treated embryos were analyzed, components involved in protein folding and ER stress were found in the AHA only replicates (Pdia6, Nap1l4, Cct3, Cct7) or showed significant upregulation when compared with PBS injected controls (Dnaja2, Ruvbl2, Psmd5, Pdia6) (Table S1). One differentially expressed protein, Pdia6, a member of the protein disulfide isomerase family, was identified in band 1 in the AHA sample only, and in band 2 as a significantly upregulated protein in AHA compared with PBS. Pdia6 is expressed during times of ER stress and is thought to help attenuate the unfolded protein response via mechanisms independent of disulfide bond forming activity^29^,^30^. However, the differential expression may be due to interspecimen variability as Pdia6 is found in the E11.5 murine lung and has been shown to be essential during *C. elegans* development^30^,^31^.

It is not unexpected that components involved in protein folding show a difference in expression between the two treatments, given the differences in side chain chemistry between Met and AHA (Fig. 1A). What is surprising is the how little overall effect AHA treatment has on the identity and quantity of proteins. This corroborates our observation that AHA treatment does not overtly disrupt embryogenesis or subsequent development after parturition.

Additional biochemical tools that have been shown to label newly synthesized proteins *in vivo* include stable isotope labeling with amino acids in cell culture (SILAC) and incorporation of puromycin derivatives into nascent proteins^19^,^32^,^33^. SILAC uses amino acids with stable isotopes that can be used to resolve newly synthesized proteins using LC-MS/MS; however, it is not possible to identify proteins in low abundance without additional costly protein/peptide fractionation. Comparative experiments have shown that SILAC-based measurements identify approximately an order of magnitude fewer newly synthesized proteins than when enriched using BONCAT^9^,^34^. Incorporation of puromycin derivatives can be used for enrichment with antibody pull-down or CuAAC^19^,^32^. A limitation of these methods is that protein synthesis stops upon puromycin incorporation, reducing the biological activity of the truncated proteins and preventing their utility for long term labeling. In contrast to these methods, BONCAT enables the enrichment of newly synthesized proteins within complex mixtures^9^,^10^,^34^ and does not overtly perturb protein synthesis levels as demonstrated *in vitro*^10^, *in vivo*^13^ and in this study.

Our successful demonstration that AHA and HPG can be directly administered *in vivo* for labeling of newly synthesized proteins will enable future studies that seek to characterize the dynamics of protein synthesis and turnover as a function of development and disease in a mammalian system.

## Materials and Methods

Unless otherwise specified, all reagents were of chemical grade from Sigma-Aldrich.

### Animal care

Wild-type C57BL6 mice were used in this study, derived from animals obtained from The Jackson Laboratory. All experimental protocols were performed in accordance with the guidelines established by the Purdue Animal Care and Use Committee, and all methods were approved by this committee (PACUC; protocol# 1209000723). PACUC ensures that all animal programs, procedures, and facilities at Purdue University adhere to the policies, recommendations, guidelines, and regulations of the USDA and the United States Public Health Service in accordance with the Animal Welfare Act and Purdue’s Animal Welfare Assurance.

### IP injection of AHA/HPG

AHA and HPG (Click Chemistry Tools) were diluted in PBS, raised to pH 7.4 with NaOH, sterilized with a 0.22 μm filter and stored at −20 **°**C. Newly weaned male and female pups (21–35 days old; 14–20 g) were injected IP daily for two days with 0.1 mg/g, 0.05 mg/g or 0.025 mg/g AHA or HPG in a volume of 50–200 μL. Females were time mated, where noon of the day of the recorded plug was considered to be E0.5 and injected with 0.1 mg/g per day AHA for two days, starting at E10.5. Mice were euthanized via CO_2_ inhalation, and samples from heart, lung, brain, skeletal muscle, kidney and whole embryos were obtained.

### BONCAT Labeling of AHA/HPG injected tissues

Tissues (10–50 mg) were homogenized in 2 mL ice cold lysis buffer (150 mM NaCl, 1% Triton X-100, 0.1% SDS and 50 mM Tris in H_2_O) using a Qiagen Tissue Ruptor and rocked at 4 **°**C for 2 hours. Lysates were centrifuged at 18,000 × g for 10 min at 4 **°**C and the soluble fraction was removed for analysis and stored at −80 °C. Protein concentration was quantified using the Pierce 660 nm Protein Assay.

AHA and HPG incorporated proteins within the lysates were labeled selectively with AF555-conjugated alkyne or azide using CuAAC, following^35^,^36^,^37^. Soluble protein lysate from AHA, HPG, and PBS samples (60 μL of 1.5–4 μg/μL lysate) was combined with 10 μL 400 mM sodium ascorbate, alkylated by the addition of 20 μL 0.5 M iodoacetamide and incubated for 5 min. The following were added, with vortexing after each addition: 0.5 μL 8 mM alkyne/azide-labeled fluorophore, 16 μL 25 mM CuSO_4_, 40 μL 50 mM tris(3-hydroxypropyltriazolylmethyl)amine (Click Chemistry Tools), and 40 μL 100 mM aminoguandine (pH 7). The final mixture was rotated end-over-end for 15 min at room temperature and protected from light. Unreacted dye was removed using methanol-chloroform precipitation and the protein pellets were air dried for at least 30 min. Pellets were resolubilized in 1X Laemmli Sample Buffer (Bio-Rad, CA) with 5% β-mercaptoethanol and boiled at 95 °C for 5 min, then desalted with 0.5 mL 7K MWCO Zeba Spin Desalting Columns (ThermoFisher). Samples were brought to a 1X concentration of Laemmli buffer and protein concentration was again determined using Pierce 660 nm Protein Assay to ensure equal loading before resolving by SDS-PAGE on 4–20% polyacrylamide gels (BioRad, CA) and imaged using an Azure Biosystems c400. Images were false-colored green to facilitate visualization. Loading consistency was confirmed by staining the gels with Coomassie Blue-based GelCode Blue Protein Stain (Pierce) after fluorescent imaging.

### LC-MS/MS analysis of embryo lysates

Proteins were size separated with SDS-PAGE and bands were carefully cut out to ensure that the same bands for AHA and PBS samples were excised with no cross contamination. Bands were subject to in gel digestion with trypsin and the resultant peptides were desalted and pre-concentrated using C_18_ StageTips. Proteomic analysis was performed with LC-MS/MS using the ABSciex Triple TOF 5600 coupled to the Eskigent Nano425 LC. Protein identification was performed using the MaxQuant and Protein Pilot algorithms along with manual validation.

## Additional Information

**How to cite this article**: Calve, S. *et al*. Incorporation of non-canonical amino acids into the developing murine proteome. *Sci. Rep.* **6**, 32377; doi: 10.1038/srep32377 (2016).

## Supplementary Material

Supplementary Information

Supplementary Table S1

## References

[b1] Choudhary C. & Mann M. Decoding signalling networks by mass spectrometry-based proteomics. Nat Rev Mol Cell Biol 11, 427–439 (2010). 20461098 10.1038/nrm2900

[b2] Sun L. . Quantitative proteomics of Xenopus laevis embryos: expression kinetics of nearly 4000 proteins during early development. Sci Rep 4, 4365 (2014). 24626130 10.1038/srep04365 PMC3953746

[b3] Peshkin L. . On the Relationship of Protein and mRNA Dynamics in Vertebrate Embryonic Development. Dev Cell 35, 383–394 (2015). 26555057 10.1016/j.devcel.2015.10.010 PMC4776761

[b4] Hartl D. . Transcriptome and proteome analysis of early embryonic mouse brain development. Proteomics 8, 1257–1265 (2008). 18283662 10.1002/pmic.200700724

[b5] Lucitt M. B. . Analysis of the zebrafish proteome during embryonic development. Mol Cell Proteomics 7, 981–994 (2008). 18212345 10.1074/mcp.M700382-MCP200 PMC2401336

[b6] Hill R. C., Calle E. A., Dzieciatkowska M., Niklason L. E. & Hansen K. C. Quantification of Extracellular Matrix Proteins from a Rat Lung Scaffold to Provide a Molecular Readout for Tissue Engineering. Mol Cell Proteomics (2015). 10.1074/mcp.M114.045260 PMC4390273 25660013

[b7] Decaris M. L. . Proteomic analysis of altered extracellular matrix turnover in bleomycin-induced pulmonary fibrosis. Mol Cell Proteomics 13, 1741–1752 (2014). 24741116 10.1074/mcp.M113.037267 PMC4083112

[b8] Geiger T. . Initial quantitative proteomic map of 28 mouse tissues using the SILAC mouse. Mol Cell Proteomics 12, 1709–1722 (2013). 23436904 10.1074/mcp.M112.024919 PMC3675825

[b9] Bagert J. D. . Quantitative, time-resolved proteomic analysis by combining bioorthogonal noncanonical amino acid tagging and pulsed stable isotope labeling by amino acids in cell culture. Mol Cell Proteomics 13, 1352–1358 (2014). 24563536 10.1074/mcp.M113.031914 PMC4014290

[b10] Dieterich D. C., Link A. J., Graumann J., Tirrell D. A. & Schuman E. M. Selective identification of newly synthesized proteins in mammalian cells using bioorthogonal noncanonical amino acid tagging (BONCAT). Proc Natl Acad Sci USA 103, 9482–9487 (2006). 16769897 10.1073/pnas.0601637103 PMC1480433

[b11] Kiick K. L., Weberskirch R. & Tirrell D. A. Identification of an expanded set of translationally active methionine analogues in Escherichia coli. FEBS Lett 502, 25–30 (2001). 11478942 10.1016/s0014-5793(01)02657-6

[b12] Rostovtsev V. V., Green L. G., Fokin V. V. & Sharpless K. B. A stepwise huisgen cycloaddition process: copper(I)-catalyzed regioselective “ligation” of azides and terminal alkynes. Angew Chem Int Ed Engl 41, 2596–2599 (2002). 12203546 10.1002/1521-3773(20020715)41:14<2596::AID-ANIE2596>3.0.CO;2-4

[b13] Hinz F. I., Dieterich D. C., Tirrell D. A. & Schuman E. M. Non-canonical amino acid labeling *in vivo* to visualize and affinity purify newly synthesized proteins in larval zebrafish. ACS Chem Neurosci 3, 40–49 (2012). 22347535 10.1021/cn2000876 PMC3278164

[b14] Agard N. J., Prescher J. A. & Bertozzi C. R. A strain-promoted [3 + 2] azide-alkyne cycloaddition for covalent modification of biomolecules in living systems. J Am Chem Soc 126, 15046–15047 (2004). 15547999 10.1021/ja044996f

[b15] Kolb H. C., Finn M. G. & Sharpless K. B. Click Chemistry: Diverse Chemical Function from a Few Good Reactions. Angew Chem Int Ed Engl 40, 2004–2021 (2001). 11433435 10.1002/1521-3773(20010601)40:11<2004::AID-ANIE2004>3.0.CO;2-5

[b16] Dieterich D. C. . *In situ* visualization and dynamics of newly synthesized proteins in rat hippocampal neurons. Nat Neurosci 13, 897–905 (2010). 20543841 10.1038/nn.2580 PMC2920597

[b17] Shen W. . Acute synthesis of CPEB is required for plasticity of visual avoidance behavior in Xenopus. Cell Rep 6, 737–747 (2014). 24529705 10.1016/j.celrep.2014.01.024 PMC3962200

[b18] Ullrich M. . Bio-orthogonal labeling as a tool to visualize and identify newly synthesized proteins in Caenorhabditis elegans. Nat Protoc 9, 2237–2255 (2014). 25167056 10.1038/nprot.2014.150

[b19] Liu J., Xu Y., Stoleru D. & Salic A. Imaging protein synthesis in cells and tissues with an alkyne analog of puromycin. Proc Natl Acad Sci U S A 109, 413–418 (2012). 22160674 10.1073/pnas.1111561108 PMC3258597

[b20] Beatty K. E. . Fluorescence visualization of newly synthesized proteins in mammalian cells. Angew Chem Int Ed Engl 45, 7364–7367 (2006). 17036290 10.1002/anie.200602114

[b21] Kiick K. L., Saxon E., Tirrell D. A. & Bertozzi C. R. Incorporation of azides into recombinant proteins for chemoselective modification by the Staudinger ligation. Proc Natl Acad Sci USA 99, 19–24 (2002). 11752401 10.1073/pnas.012583299 PMC117506

[b22] McClatchy D. B. . Pulsed Azidohomoalanine Labeling in Mammals (PALM) Detects Changes in Liver-Specific LKB1 Knockout Mice. J Proteome Res 14, 4815–4822 (2015). 26445171 10.1021/acs.jproteome.5b00653 PMC4642245

[b23] Schiapparelli L. M. . Direct detection of biotinylated proteins by mass spectrometry. J Proteome Res 13, 3966–3978 (2014). 25117199 10.1021/pr5002862 PMC4156236

[b24] Dayal S. . Endothelial dysfunction and elevation of S-adenosylhomocysteine in cystathionine beta-synthase-deficient mice. Circ Res 88, 1203–1209 (2001). 11397788 10.1161/hh1101.092180

[b25] Elmore C. L. . Metabolic derangement of methionine and folate metabolism in mice deficient in methionine synthase reductase. Mol Genet Metab 91, 85–97 (2007). 17369066 10.1016/j.ymgme.2007.02.001 PMC1973089

[b26] Chapman M. E., Hu L., Plato C. F. & Kohan D. E. Bioimpedance spectroscopy for the estimation of body fluid volumes in mice. Am J Physiol Renal Physiol 299, F280–283 (2010). 20462974 10.1152/ajprenal.00113.2010 PMC2904176

[b27] Troen A. M., Lutgens E., Smith D. E., Rosenberg I. H. & Selhub J. The atherogenic effect of excess methionine intake. Proc Natl Acad Sci USA 100, 15089–15094 (2003). 14657334 10.1073/pnas.2436385100 PMC299913

[b28] van Hest J. C. & Tirrell D. A. Efficient introduction of alkene functionality into proteins *in vivo*. FEBS Lett 428, 68–70 (1998). 9645477 10.1016/s0014-5793(98)00489-x

[b29] Vekich J. A., Belmont P. J., Thuerauf D. J. & Glembotski C. C. Protein disulfide isomerase-associated 6 is an ATF6-inducible ER stress response protein that protects cardiac myocytes from ischemia/reperfusion-mediated cell death. J Mol Cell Cardiol 53, 259–267 (2012). 22609432 10.1016/j.yjmcc.2012.05.005 PMC3423192

[b30] Eletto D., Eletto D., Dersh D., Gidalevitz T. & Argon Y. Protein disulfide isomerase A6 controls the decay of IRE1alpha signaling via disulfide-dependent association. Mol Cell 53, 562–576 (2014). 24508390 10.1016/j.molcel.2014.01.004 PMC3977204

[b31] Liu Y. & Hogan B. L. Differential gene expression in the distal tip endoderm of the embryonic mouse lung. Gene Expr Patterns 2, 229–233 (2002). 12617806 10.1016/s1567-133x(02)00057-1

[b32] Goodman C. A. . Novel insights into the regulation of skeletal muscle protein synthesis as revealed by a new nonradioactive *in vivo* technique. FASEB J 25, 1028–1039 (2011). 21148113 10.1096/fj.10-168799 PMC3042844

[b33] Ong S. E. . Stable isotope labeling by amino acids in cell culture, SILAC, as a simple and accurate approach to expression proteomics. Mol Cell Proteomics 1, 376–386 (2002). 12118079 10.1074/mcp.m200025-mcp200

[b34] Zhang G. . In-depth quantitative proteomic analysis of de novo protein synthesis induced by brain-derived neurotrophic factor. J Proteome Res 13, 5707–5714 (2014). 25271054 10.1021/pr5006982 PMC4261974

[b35] van Geel R., Pruijn G. J., van Delft F. L. & Boelens W. C. Preventing thiol-yne addition improves the specificity of strain-promoted azide-alkyne cycloaddition. Bioconjug Chem 23, 392–398 (2012). 22372991 10.1021/bc200365k

[b36] Tornoe C. W., Christensen C. & Meldal M. Peptidotriazoles on solid phase: [1,2,3]-triazoles by regiospecific copper(i)-catalyzed 1,3-dipolar cycloadditions of terminal alkynes to azides. J Org Chem 67, 3057–3064 (2002). 11975567 10.1021/jo011148j

[b37] Hong V., Presolski S. I., Ma C. & Finn M. G. Analysis and optimization of copper-catalyzed azide-alkyne cycloaddition for bioconjugation. Angew Chem Int Ed Engl 48, 9879–9883 (2009). 19943299 10.1002/anie.200905087 PMC3410708

