# Supplementary material for: Incorporation of non-canonical amino acids into the developing murine proteome
Source: Sci Rep. 2016 Aug 30;6:32377. doi: 10.1038/srep32377 (PMC5004113; doi:10.1038/srep32377)
Supplement: Supplementary Information [file srep32377-s1.pdf]

Supplemental Information

Incorporation of non-canonical amino acids into the developing murine proteome

Sarah Calve, Andrew Witten, Alexander Ocken and Tamara L. Kinzer-Ursem

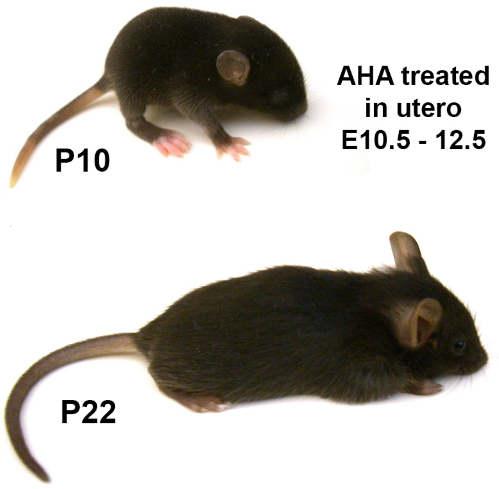

Figure S1. AHA administration *in utero* does not affect development after parturition. When allowed to come to term, pups at P10 and P22 had reached the appropriate milestones, where at P10 the eyes were still closed but there was fur on the belly. At P22, the pups were active and showed no overt developmental delays.
